# Pro-adrenomedullin as prognostic biomarker in the sepsis

**DOI:** 10.1186/cc11971

**Published:** 2013-03-19

**Authors:** M De La Torre-Prados, A Garcia-De la Torre, A Enguix, M Mayor, N Zamboschi, C Trujillano-Fernández, A Garcia-Alcantara

**Affiliations:** 1 Hospital Virgen de la Victoria, Málaga, Spain

## Introduction

Measurement of biomarkers is a potential approach to early assessment and prediction of mortality in septic patients. The purpose of this study was to ascertain the prognostic value of pro-adrenomedullin (pADM), measured in all patients admitted to the ICU of our hospital with a diagnosis of severe sepsis or septic shock during 1 year.

## Methods

A cohort study of 117 patients >18 years with severe sepsis according to the Surviving Sepsis Campaign, in an ICU of a university hospital. Demographic, clinical parameters and pADM, C-reactive protein and procalcitonin were studied during 1year. Descriptive and comparative statistical analysis was performed using the statistical software packages Statistica Stat Soft Inc 7.1 and MedCalc 9.2.1.0.

## Results

We analyzed 117 consecutive episodes of severe sepsis (15%) or septic shock (85%) in the ICU. The median age of the patients was 64 (interquartile range, 53 to 72) years; the main sources of infection were respiratory tract (46%) and intra-abdomen (21%). The 28-day mortality was 32.5%. The profile of death patients had a significantly higher average age (64.7 vs. 57.6 years; *P *= 0.024), as well as clinical severity scores, APACHE II (26.6 vs. 23; *P *= 0.006) and SOFA (11.6 vs. 89.2; *P <*0.001). Kaplan-Meier survival analysis was significant. *P *= 0.0017 for patients with pADM <1.2 nmol/l. Cox regression analysis also showed statistical significance (*P *= 0.0033) and a likelihood ratio = 1.18 per each 1 nmol/l increase in pADM.

## Conclusion

The protein pADM is an important prognostic biomarker of survival when measured on admission of septic patients to the ICU.
